# Case Report: CD20/BCMA bispecific CAR-T therapy for progressive multiple sclerosis

**DOI:** 10.3389/fimmu.2026.1965459

**Published:** 2026-09-29

**Authors:** Zhenfen Ling, Xia Liu, Wensi Li, Qingguo Wang, Nu Tan, Xueying Ding, Yunyi Zan, Youyao Gu, Yuan Qian, Xiaobing Luo, Thule Trinh, Yanfeng Li, Xiaoteng Lv, Minna Wang, Qian Tan, Kevin Zhu, Chengxiao Zheng, Xin Yao, Judy Zhu, Jiaqi Huang, Yihong Yao, Nan Shen

**Affiliations:** 1 Shanghai General Hospital, Shanghai, China; 2 Shanghai AbelZeta Ltd., Shanghai, China; 3 AbelZeta Inc., Rockville, MD, United States; 4 The University of Vermont Medical Center, Burlington, VT, United States; 5 Renji Hospital, Shanghai Jiao Tong University School of Medicine, Shanghai, China

**Keywords:** CAR-T therapy, case report, CD20/BCMA, drug-free remission, secondary progressive multiple sclerosis

## Abstract

Refractory secondary progressive multiple sclerosis (SPMS) remains an area of unmet need, particularly once disability continues to accumulate despite multiple disease-modifying therapies. Two SPMS patients are reported with C-CAR168 treatment, a bispecific CD20/BCMA CAR-T product, achieving 12- and 6-month drug-free remission. Patient 001 (EDSS 4.0→3.0) showed improvement of oculomotor dysfunction, ataxia, and cognitive impairment. Patient 002 (EDSS 6.5→6.0) demonstrated halted lower-limb progression and relieved bowel/bladder dysfunction. C-CAR168 infiltrated into the cerebrospinal fluid (CSF), persistently depleted pathogenic B-lineage cells and CD20dim T cells, and normalized inflammatory markers with an overall favorable safety profile. This first-in-human experience supports bispecific CAR-T as a promising immune-reprogramming strategy for refractory SPMS.

## 1 Introduction

Secondary progressive multiple sclerosis (SPMS) is a clinical phenotype of multiple sclerosis (MS) characterized by characterized by ongoing disability accumulation that is inadequately controlled by current disease-modifying therapies (DMTs), particularly in non-active SPMS (1, 2). Current peripherally acting immunosuppressants, including anti-CD20 monoclonal antibodies and S1P receptor modulators, fail to efficiently penetrate the blood-brain barrier and therefore incompletely target compartmentalized CNS-resident B-lineage cells and plasma cells, necessitating continuous administration that carries cumulative risks of hypogammaglobulinemia, infection, and hematologic adverse events (3). Optic-spinal form of multiple sclerosis (OSMS) accounts for a higher proportion of MS populations in Asian than in Western countries, with a significant more frequent longitudinally extensive spinal cord lesions (LESCLs) on Magnetic Resonance Imaging (MRI) resulting in more severe clinical manifestations and poorer response to traditional immunomodulator such as Interferon-beta (4–6). Although siponimod and ocrelizumab confer benefits in delaying disability progression in progressive MS, no currently approved therapy can halt neurodegeneration or restore function (1, 2).Chimeric antigen receptor T (CAR-T) cell therapy is undergoing early exploration in autoimmune disease, including multiple sclerosis, and is mechanistically attractive in SPMS given the established importance of B-cell biology to disease pathophysiology and the potential to more deeply deplete pathogenic B-cell and plasma-cell compartments than existing therapies (7, 8). A dual-target approach simultaneously engaging CD20+ B cells and BCMA+ plasma cells may achieve more complete immune reprogramming and drug-free remission—an unmet goal in refractory SPMS. Here we describe the first clinical experience with C-CAR168, an autologous bispecific CD20/BCMA CAR-T therapy, in two patients with refractory SPMS enrolled in the phase 1 CAR-AID study (9) (NCT06249438).

## 2 Case presentation

### 2.1 Patient information

Patient 001 was a 31-year-old man diagnosed with MS in 2014. His disease followed a relapsing-remitting course, until early 2024, when he developed confirmed disability progression and met the clinical criteria for SPMS. Prior therapies included glucocorticoid pulses, interferon β-1b, teriflunomide, and off-label azathioprine (**Table 1**), all failing to halt progression. At baseline, he exhibited recurrent diplopia, gait difficulty, imbalance, cognitive symptoms, with newly onset T1 lesions on MRI.

**Table 1:** Patient demographics.

Characteristic	Patient 001	Patient 002
Age, year	31	35
Gender	Male	Female
Type of MS	SPMS	SPMS
Time since initial diagnosis, years	10.25	4.25
Prior treatment	Glucocorticoid pulses, Interferon β-1b, Teriflunomide, Azathioprine	Glucocorticoid pulses, IVIg, Ofatumumab, Ozanimod
Clinical manifestations	Recurrent diplopia, gait difficulty, balance impairment, cognitive symptoms	Progressive lower-limb weakness, numbness, spasticity, and gait instability
EDSS score	4.0	6.5
NfL concentration, pg/ml	97.47	4.16
CSF protein, mg/L	0.55	0.38
IgG in CSF, mg/L	41.1	33.5

MS, multiple sclerosis; SPMS, secondary-progressive multiple sclerosis; IVIg, intravenous immunoglobulin; EDSS, expanded disability status scale; NfL, neurofilament light chain; CSF, cerebrospinal fluid; IgG, immunoglobulin G.

Patient 002 was a 35-year-old woman with SPMS and progressive lower-limb weakness, numbness, spasticity, and gait instability. She had failed intravenous immunoglobulin, glucocorticoid pulses, ofatumumab, and ozanimod (**Table 1**). At baseline, she required bilateral assistance to walk ≥20 m.

Both patients fulfilled the 2017 McDonald diagnostic criteria (10), had evidence of disease on screening/baseline MRI, and met the study’s definition of refractory MS, which required ongoing disease activity or relapses or progression despite prior standard treatment with at least two DMTs, each administered for a standard course of at least 8 weeks before screening, and provided written informed consent.

### 2.2 Clinical findings

Patient 001 at baseline showed ocular misalignment with left gaze preference, impaired right eye adduction, horizontal nystagmus, mild vertical gaze restriction, broad-based ataxic gait, hyperactive palmomental reflexes, mild cognitive impairment and intermittent urinary incontinence. EDSS was 4.0, mean 9-HPT 36.26 seconds, mean T25-FW 7.6 seconds, and MMSE 24. Serum NfL was markedly elevated at 97.47 pg/mL; CSF IgG was mildly elevated with normal IgG index; type II OCBs persisted; AQP4-IgG was negative. MRI showed extensive burden including large pontine lesions, new periventricular lesions, enlarging thalamic lesions, cerebral atrophy, and extensive cervical/thoracic cord involvement (**Figure 1**). MRI examinations were performed on Siemens Vida 3.0 T scanner. Slice thickness was set to 4mm for brain and 2.4mm for spinal cord and optic nerve sequences.

**Figure 1:**
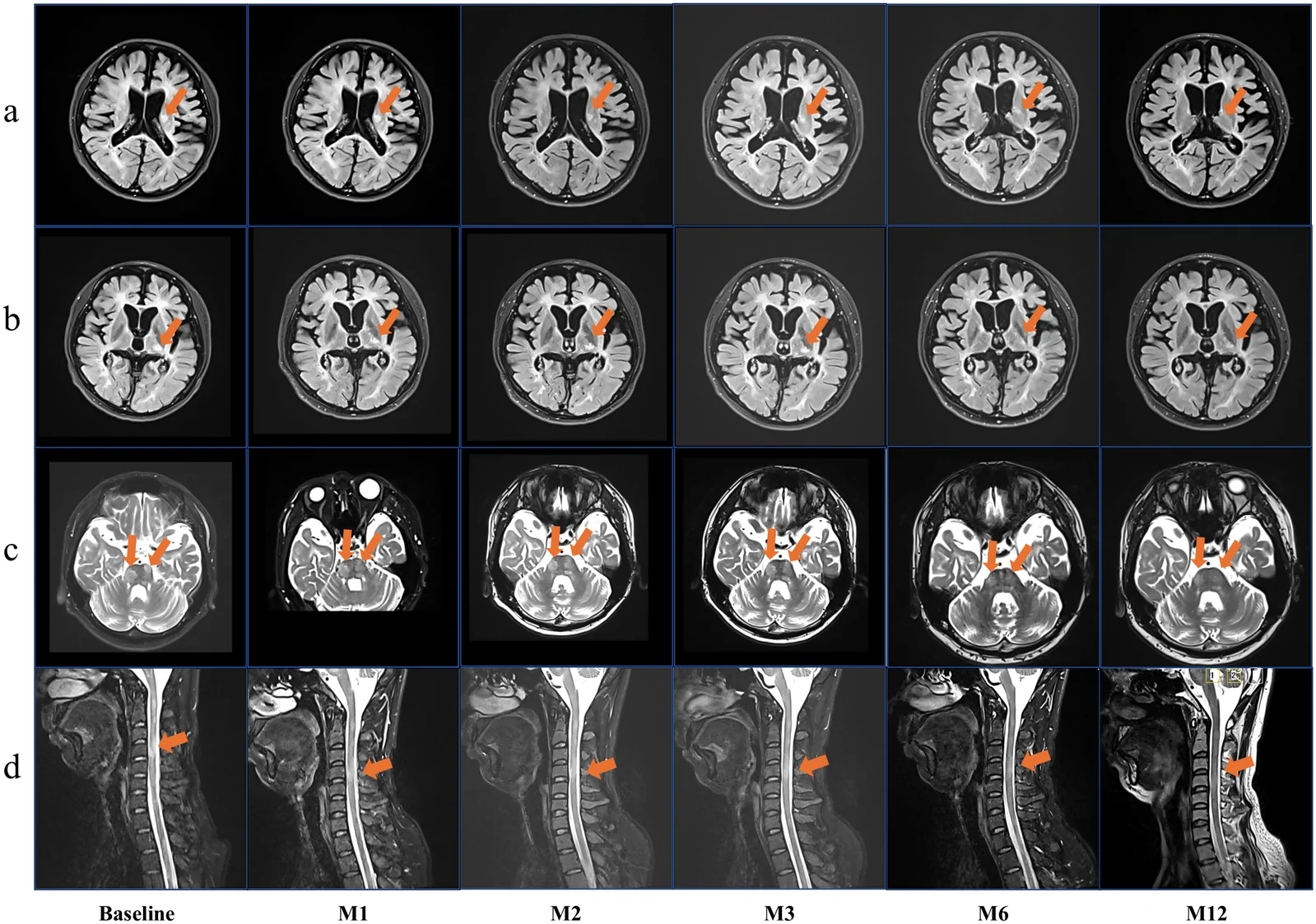
Serial MRI of the brain and spinal cord from baseline to month 12 in Patient 001. Orange arrows highlight atrophy or improvement of representative T2-weighted lesions: **(a)** lateral ventricles lesions (axial) show progressive reduction in size and decreased signal intensity on T2-FLAIR following treatment, **(b)** thalamus lesions (axial) demonstrate gradual reduction in lesion extent and signal attenuation on T2-FLAIR after treatment, **(c)** pons lesions (axial) at the identical anatomical slice display progressive shrinkage and reduced signal intensity on T2WI. Marked reduction is observed at 12-month follow-up for newly-onset bilateral pontine lesions pre-treatment, **(d)** cervical spinal cord (sagittal) lesions show decreased signal intensity, reduced lesion extent, and ameliorated edema on T2WI.

Patient 002 exhibited increased tone in bilateral lower limbs (left > right) and left upper limb, hyperreflexia, scissors gait, Babinski and Hoffman signs, and proximal-predominant weakness (left lower limb > others). MRI showed multifocal ventricular and subcortical white-matter lesions, cerebral atrophy, and a long-segment cervical cord lesion extending into the thoracic cord. EDSS 6.5, mean 9-HPT 24.46 seconds, mean T25-FW 24.2 seconds, MMSE 27, and visual acuity 0.63 OD and 0.8 OS. Serum NfL was 4.16 pg/mL (normal range); CSF protein 0.38 g/L (normal); CSF OCBs previously positive; AQP4-IgG negative.

### 2.3 Diagnosis and differential diagnosis

In both patients, the diagnosis of MS was established according to the 2017 McDonald criteria. Their classification as SPMS was based on evidence of disability progression independent of relapses, lasting for more than one year. AQP4-IgG testing excluded neuromyelitis optica spectrum disorders.

### 2.4 Treatment intervention

Both patients underwent leukapheresis followed by lymphodepletion with fludarabine 25 mg/m²/day and cyclophosphamide 300 mg/m²/day on days −5 to −3 and a single intravenous infusion of C-CAR168 at 0.75×10^6^ CAR-T cells/kg on day 0. C-CAR168 is an autologous anti-CD20/BCMA bispecific CAR-T product designed to simultaneously target B cells and autoantibody-producing plasma cells.

### 2.5 Follow-up and outcomes

Clinical symptoms improved markedly within 2 weeks post-infusion in both patients, with rapid reduction of acute lesions followed by gradual higher neurological function improvement. They both achieved drug-free remission for 12 months and 6 months, respectively, that is, no DMT, corticosteroids, or other immunosuppressants were administered during follow-up.

In Patient 001, EDSS improved from 4.0 to 3.5 at month 1 and stabilized at 3.0 through month 12. Urinary incontinence, vertical nystagmus, and ataxia resolved completely by month 3; only mild right eye adduction deficit persisted at month 12. Functional assessments (9-HPT, T25-FW, MMSE) showed continuous improvement (**Figure 2**), in which 22% improvement in T25-FW and 30% improvement in 9-HPT and the patient regained normalized imbalance, gait and running ability by month 6.5.

**Figure 2:**
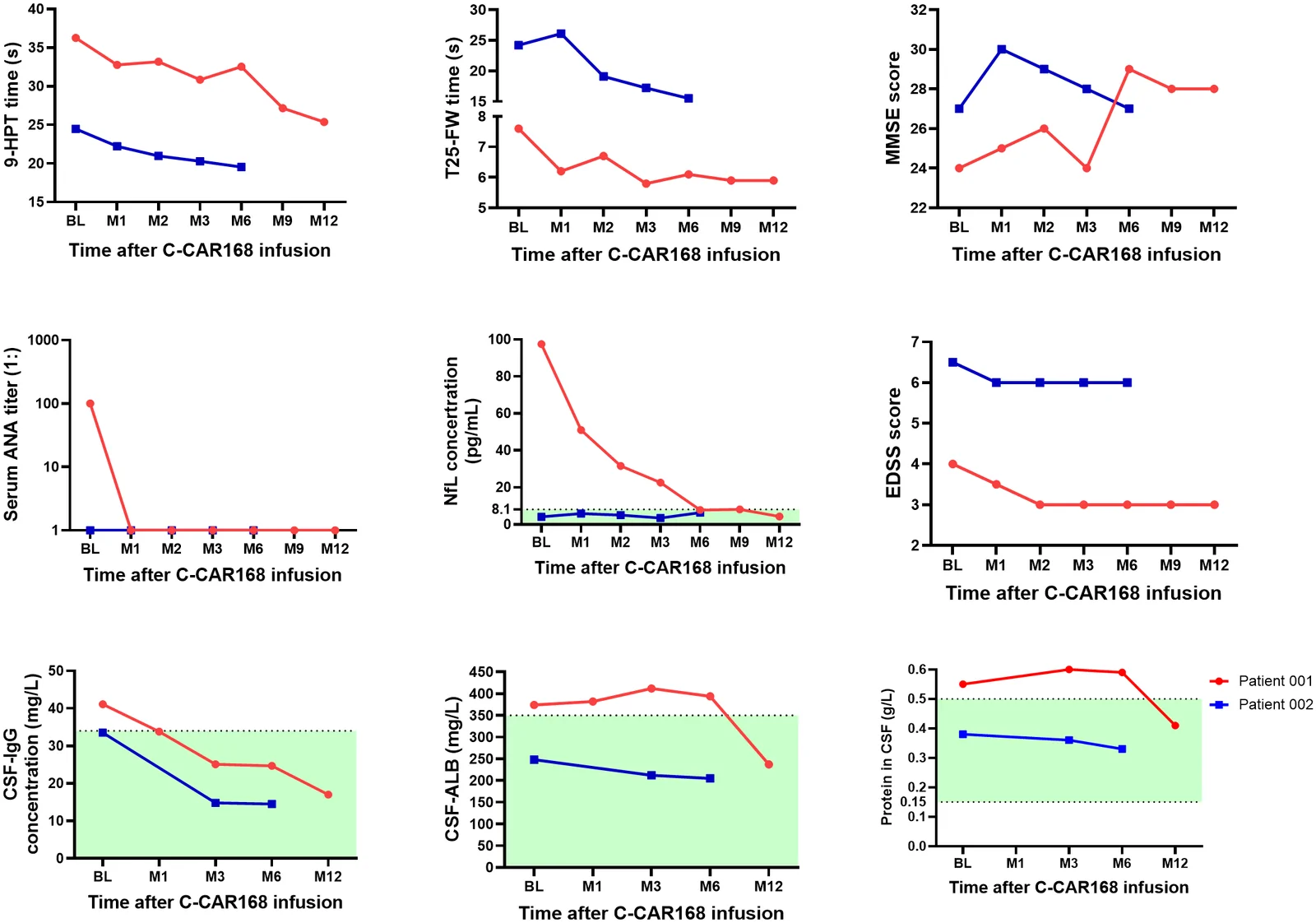
Clinical and biomarker outcomes. 9-HPT, T25-FW, MMSE, serum ANA, NfL kinetics, EDSS trajectory, and IgG, ALB and Protein in CSF.

In patient 002, EDSS improved from 6.5 at baseline to 6.0 at month 1 and remained stable at 6.0 through month 6 with halted progressive lower limb weakness, spasticity and gait instability were ameliorated, bowel and bladder incontinence were relieved, and posture as well as muscle tone improved, with previously rigid limbs becoming supple. She got 7.7% improvement in T25-FW and 36% improvement in 9-HPT. No further neurological deterioration was observed throughout the follow-up.

Serial MRI in Patient 001 showed atrophy or improvement of representative T2-weighted lesions in the periventricular region, thalamus, pons, and cervical spinal cord (**Figure 1**). No new gadolinium-enhancing lesions and no new or enlarging T2 lesions were identified by either investigator or independent review. In Patient 002, serial MRI also suggested early improvement in ventricular and cervical cord lesions.

Biomarker analysis revealed robust C-CAR168 expansion in blood and CSF, deep depletion of CD20+ B cells, plasma cells, and CD20dim T cells, and predominantly naive B-cell reconstitution (**Figure 3**). Serum NfL in Patient 001 fell from 97.47 to 4.33 pg/mL by month 12. CSF protein and IgG declined; antinuclear antibody (ANA) became negative. OCBs remained type II positive in both patients. Biomarker support in Patient 002 was more limited but directionally similar: serum IgG declined without IVIg, serum NfL and CSF protein remained within the normal range. Serum IgG, IgM, and IgA declined rapidly and bottomed within 3 months, recovering in the sequence of IgM, IgA, and IgG.

**Figure 3:**
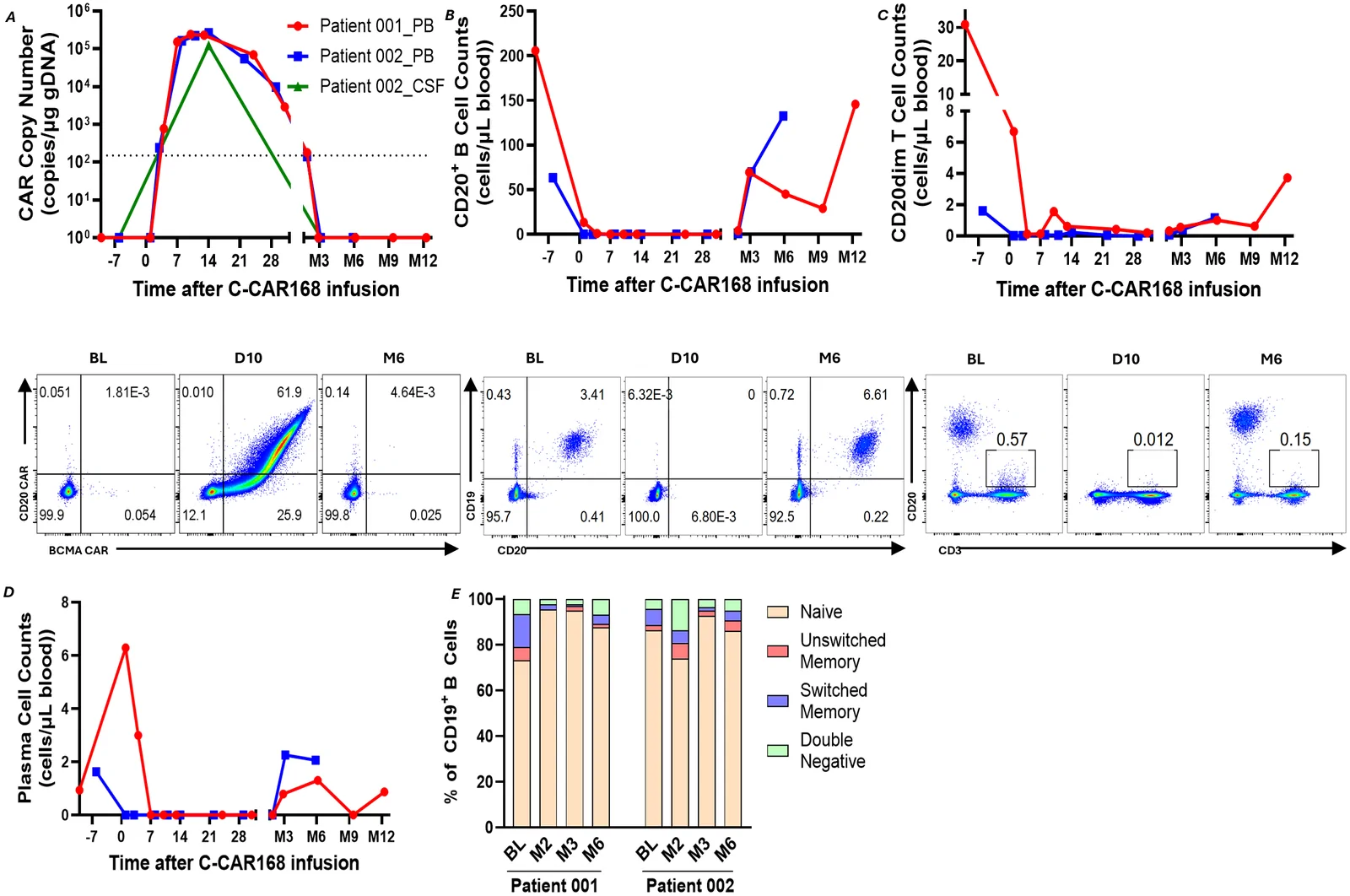
Pharmacokinetics and pharmacodynamics of C-CAR168. **(A)** qPCR kinetics in blood and CSF; flow cytometry detection in peripheral blood. **(B)** CD20+ B-cell counts and flow cytometry. **(C)** CD20dim T-cell counts and flow cytometry. **(D)** Plasma cell (CD45+BCMA+CD38bright) counts. **(E)** B-cell phenotype (IgD/CD27) reconstitution pattern.

### 2.6 Safety

By March 31, 2026, both patients maintained favorable safety profiles with no grade ≥3 treatment-related adverse events or SAEs. Only patient 001 developed grade 1 CRS, resolved within 4 days without tocilizumab or corticosteroids. No immune effector cell-associated neurotoxicity syndrome (ICANS) was observed in either patient. Treatment-related adverse events were limited to grade 1 neutrophil count decreased, hypertransaminasemia, pyrexia and hypogammaglobulinemia, all manageable with supportive care. Patient 001 had grade 1 nasopharyngitis at month 2 (spontaneous recovery); Patient 002 had grade 1 upper respiratory tract infection at month 6. Both events were considered unrelated to C-CAR168. Although both patients showed post-infusion immunoglobulin decline, neither patient required IVIg during follow-up. We also observed that protective antibodies against measles, mumps, and VZV remained seropositive without seroreversion despite deep B-cell depletion. In addition, dose escalation to 1.5×10^6^ CAR-T cells/kg was implemented in the CAR-AID study, and showed good tolerability.

## 3 Discussion

This report provides the first clinical evidence that bispecific CD20/BCMA CAR-T induces drug-free remission in refractory SPMS. Two prior literature reports in MS employed single-target approaches: CD19 CAR-T in two patients with relapsing-remitting MS showed clinical improvement but required closer monitoring for efficacy durability (7); and anti-BCMA CAR-T demonstrated deep plasma-cell depletion (8). Our dual-target strategy conceptually extends both by simultaneously engaging the B-cell and plasma-cell compartments, potentially explaining the robust clinical response observed here.

The drug-free remission for up to 12 months without maintenance immunosuppression represents a distinct advantage over conventional therapies requiring lifelong administration (1, 2). The combination of clinical improvement, functional measures (EDSS, 9-HPT, T25-FW, MMSE), MRI and biomarker trend strengthens the signal beyond any single endpoint. Autoreactive T and B cells cross the blood–brain barrier (BBB), triggering inflammatory demyelination and axonal injury in MS patients (11). The mechanism may involve profound B-lineage resetting: C-CAR168 infiltrates the CNS, persistently depletes pathogenic B cells and CD20dim T cells, and normalizes intrathecal inflammation, since CD20dim T cells are reported to produce a proinflammatory subset that infiltrates the CNS and exacerbates tissue damage (3, 12). The dissociation between persistent type II OCBs and favorable outcomes corroborates emerging evidence that some intrathecal OCBs are secondary biomarkers of tissue injury rather than direct pathogenic drivers (13, 14). Preserved protective antibody titers despite deep B-cell depletion, which is a favorable safety signal.

We also noted that patient 002 had normal baseline NfL despite higher EDSS and more advanced disability. That aligns with existing evidence that serum NfL primarily reflects the rate of axonal injury over the recent weeks to months, rather than the cumulative irreversible neurodegeneration or fixed disability (15) NfL may be a less informative biomarker for treatment response in Patient 002 specifically.

These cases are best interpreted within the Lublin 2014 framework, in which SPMS may remain active or non-active and ongoing inflammatory activity does not exclude a progressive phenotype (16). Given that the progressive clinical course since 2024 and new lesions in MRI of patient 001, active SPMS was considered. In patient 002, she got progressive clinical course since beginning but no new signs showed in MRI at baseline, non-active SPMS was considered. This report is limited by the small sample size (n=2) and shorter follow-up in Patient 002. Single-center experience limits generalizability, although temporal association and biomarker convergence strongly support on-target effects. Longer observation is needed to define durability of immune recovery and infection risk. If confirmed in larger trials, single-dose bispecific CAR-T could transform the treatment paradigm for refractory SPMS by offering off-drug remission after a one-time intervention. Serial CSF immune markers may serve as practical readouts for CNS treatment response in future neuroimmunology cell therapy studies.

## 4 Patient perspective

Both patients reported substantial subjective improvement complementing objective measures.

Patient 001 described regaining confidence in daily activities: “I can walk steadily without fearing a fall, I can even run, and my vision is clear again. I feel like a completely different person.” Patient 002 noted: “My legs no longer feel like stone; I have no more painful spasms, can attempt climbing stairs, and find it easier than before.” Neither patient reported feeling burdened by the single infusion, both preferring it over previous regimens requiring repeated administrations. These subjective accounts reinforce the functional and quality-of-life gains.

## Data Availability

The original contributions presented in the study are included in the article/Supplementary Material. Further inquiries can be directed to the corresponding authors.

## References

[1] Thompson AJ Baranzini SE Geurts J Hemmer B Ciccarelli O . Multiple sclerosis. Lancet. (2018) 391:1622–36. doi: 10.1016/S0140-6736(18)30481-1 29576504

[2] Jakimovski D Bittner S Zivadinov R Morrow SA Benedict RH Zipp F . Multiple sclerosis. Lancet. (2024) 403:183–202. doi: 10.1016/S0140-6736(23)01473-3 37949093

[3] Zierfuss B Larochelle C Prat A . Blood-brain barrier dysfunction in multiple sclerosis: causes, consequences, and potential effects of therapies. Lancet Neurol. (2024) 23:95–109. doi: 10.1016/S1474-4422(23)00377-0 38101906

[4] Li TR Xiao H Li SJ Du XK Zhou J . Multiple sclerosis: clinical features and MRI findings in Northern China. Eur J Med Res. (2014) 19:20. doi: 10.1186/2047-783X-19-20 24731721 PMC4003518

[5] Matsuoka T Matsushita T Osoegawa M Ochi H Kawano Y Mihara F . Heterogeneity and continuum of multiple sclerosis in Japanese according to magnetic resonance imaging findings. J Neurol Sci. (2008) 266:115–25. doi: 10.1016/j.jns.2007.09.010 17950755

[6] Wang KC Lin KH Lee TC Lee CL Chen SY Chen SJ . Poor responses to interferon-beta treatment in patients with neuromyelitis optica and multiple sclerosis with long spinal cord lesions. PloS One. (2014) 9:e98192. doi: 10.1371/journal.pone.0098192 24887452 PMC4041653

[7] Fischbach F Richter J Pfeffer LK Fehse B Berger SC Reinhardt S . CD19-targeted chimeric antigen receptor T cell therapy in two patients with multiple sclerosis. Med. (2024) 5:550–558.e2. doi: 10.1016/j.medj.2024.03.002 38554710

[8] Qin C Dong MH Zhou LQ Chu YH Pang XW He JY . Anti-BCMA CAR T-cell therapy in patients with multiple sclerosis. Cell. (2025) 188:6414–6423.e11. doi: 10.1016/j.cell.2025.09.020 41101309

[9] Ding HH Li WS Shen YW Zhang CY Ye Y Wang R . Anti-CD20/BCMA bispecific CAR-T cell therapy promotes immune reset and sustained drug free remission in refractory autoimmune diseases [abstract. Arthritis Rheumatol. (2025) 77. Available online at: https://acrabstracts.org/abstract/anti-cd20-bcma-bispecific-car-t-cell-therapy-promotes-immune-reset-and-sustained-drug-free-remission-in-refractory-autoimmune-diseases/

[10] Thompson AJ Banwell BL Barkhof F Carroll WM Coetzee T Comi G . Diagnosis of multiple sclerosis: 2017 revisions of the McDonald criteria. Lancet Neurol. (2018) 17:162–73. doi: 10.1016/S1474-4422(17)30470-2 29275977

[11] Murúa SR Farez MF Quintana FJ . The immune response in multiple sclerosis. Annu Rev Pathol. (2022) 17:121–39. doi: 10.1146/annurev-pathol-052920-040318 34606377

[12] Mazzeo AC Calabresi L Damato V Spagni G Massacesi L Mariottini A . CD20+ T cells in multiple sclerosis: from pathogenesis to treatment-induced depletion. Int J Mol Sci. (2025) 26:6655. doi: 10.3390/ijms26146655 40724904 PMC12294675

[13] Comi G Bar-Or A Lassmann H Uccelli A Hartung HP Montalban X . Role of B cells in multiple sclerosis and related disorders. Ann Neurol. (2021) 89:13–23. doi: 10.1002/ana.25927 33091175 PMC8007167

[14] Kennedy PGE George W Yu XL . The elusive nature of the oligoclonal bands in multiple sclerosis. J Neurol. (2024) 271:116–24. doi: 10.1007/s00415-023-12081-7 37945762

[15] Caminero AB Fernández O . Serum neurofilament light chain in multiple sclerosis: from biological signal to clinically informed decision-making. Front Neurol. (2026) 17:1815022. doi: 10.3389/fneur.2026.1815022 42422214 PMC13341485

[16] Lublin FD Reingold SC Cohen JA Cutter GR Sørensen PS Thompson AJ . Defining the clinical course of multiple sclerosis: the 2013 revisions. Neurology. (2014) 83:278–86. doi: 10.1212/WNL.0000000000000560 24871874 PMC4117366

